# Supplementary material for: Sulforaphane exhibits antiviral activity against pandemic SARS-CoV-2 and seasonal HCoV-OC43 coronaviruses in vitro and in mice
Source: Commun Biol. 2022 Mar 18;5:242. doi: 10.1038/s42003-022-03189-z (PMC8933402; doi:10.1038/s42003-022-03189-z)
Supplement: Supplementary file 1 — Supplementary Information [file 42003_2022_3189_MOESM1_ESM.pdf]

Supplementary Information

Sulforaphane exhibits antiviral activity against pandemic SARS-CoV-2 and seasonal HCoV-OC43 coronaviruses in vitro and in mice

Alvaro A. Ordonez[1,2]*, C. Korin Bullen[2,3], Andres F. Villabona-Rueda[4], Elizabeth A. Thompson[5,6], Mitchell L. Turner[1,2], Vanessa F. Merino[7], Yu Yan[7], John Kim[2], Stephanie L. Davis[2,3], Oliver Komm[2,3], Jonathan D. Powell[5,6], Franco R. D'Alessio[4], Robert H. Yolken[8], Sanjay K. Jain[1,2], Lorraine Jones-Brando[8]*

[1] Division of Infectious Diseases, Department of Pediatrics, Johns Hopkins University School of Medicine, Baltimore, MD, USA
[2] Center for Tuberculosis Research, Johns Hopkins University School of Medicine, Baltimore, MD, USA
[3] Division of Infectious Diseases, Department of Medicine, Johns Hopkins University School of Medicine, Baltimore, MD, USA
[4] Division of Pulmonology, Department of Medicine, Johns Hopkins University School of Medicine, Baltimore, MD, USA
[5] Department of Oncology, Johns Hopkins University School of Medicine, Baltimore, MD, USA
[6] Bloomberg-Kimmel Institute for Cancer Immunotherapy, Johns Hopkins University School of Medicine, Baltimore, MD, USA
[7] Russell H. Morgan Department of Radiology and Radiological Sciences, Johns Hopkins University School of Medicine, Baltimore, MD, USA
[8] Stanley Division of Developmental Neurovirology, Department of Pediatrics, Johns Hopkins University School of Medicine, Baltimore, MD, USA

*Co-corresponding authors: aordone2@jhmi.edu (AAO) and lbrando@jhmi.edu (LJ-B)

Supplementary Figure 1. Antiviral effects of SFN against SARS-CoV-2 variants. Effects of SFN evaluated in Vero C1008 cells exposed to drug for 1 hour followed by viral inoculation. Delta and Omicron variants of SARS-CoV-2 were evaluated for CPE using a bioluminescence readout. Antiviral data is displayed in red; anti-host cell activity (cytotoxicity) is displayed in blue.

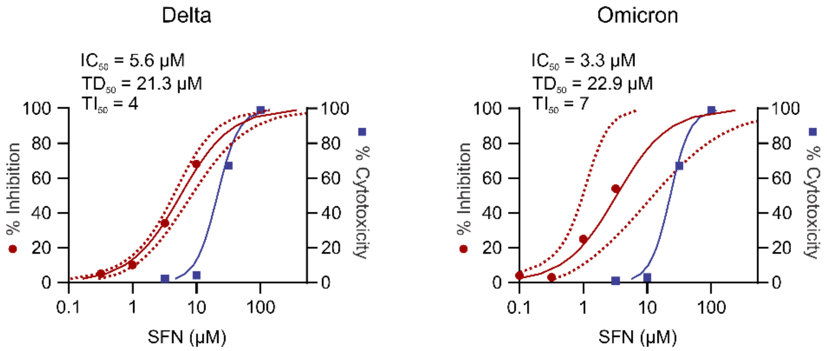

Supplementary Figure 2. Antiviral activity of SFN on *NRF2* knockdown (KD) cells. *NRF2* was silenced from Caco-2 cells by CRISPR/Cas9 to reduce the expression of NRF2. (**a**) Western blot determination of NRF2, in total cell lysate of Caco-2 cells, control, and *NRF2* KD, treated as described above. β-actin was used as loading control. (**b**) Control and *NRF2* KD cells were treated with SFN (5 μM) or DMSO (vehicle control) over 1 - 2 h. Subsequently, the cells were infected with SARS-CoV-2/USA-WA1/2020 and incubated over 48 h. Culture supernatants were collected and processed for quantification of SARS-CoV-2 copies by qRT-PCR. A significant reduction in viral load was observed in cells treated with SFN in both control and *NRF2* KD cells ($P < 0.001$). There was no significant difference in the viral load with or without SFN treatment in *NRF2* KD cells compared to control cells ($P \geq 0.54$). Data representative of three independent experiments. Statistical comparisons were made with one-way ANOVA with Tukey's multiple comparisons test.

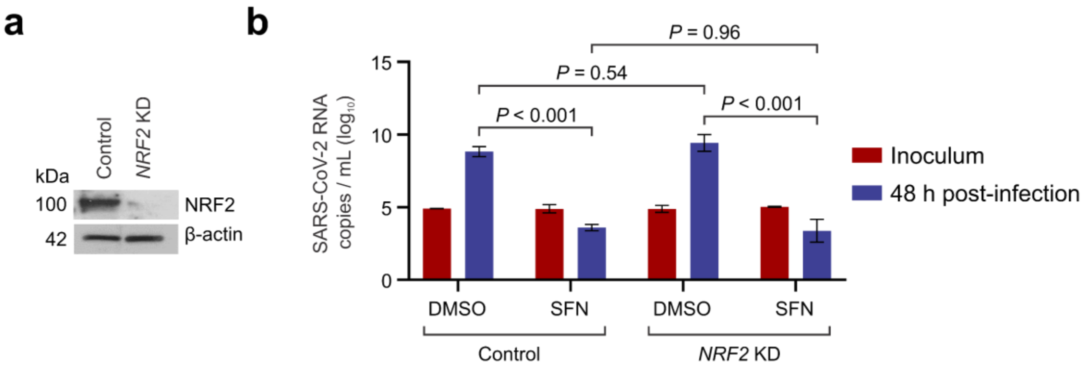

Supplementary Figure 3. hACE2 expression in lung tissues. The expression of hACE2 in the lung of K18-hACE2 mice infected with SARS-CoV-2 and treated with SFN was primarily in the airway epithelia. The lung section of representative mouse is shown.

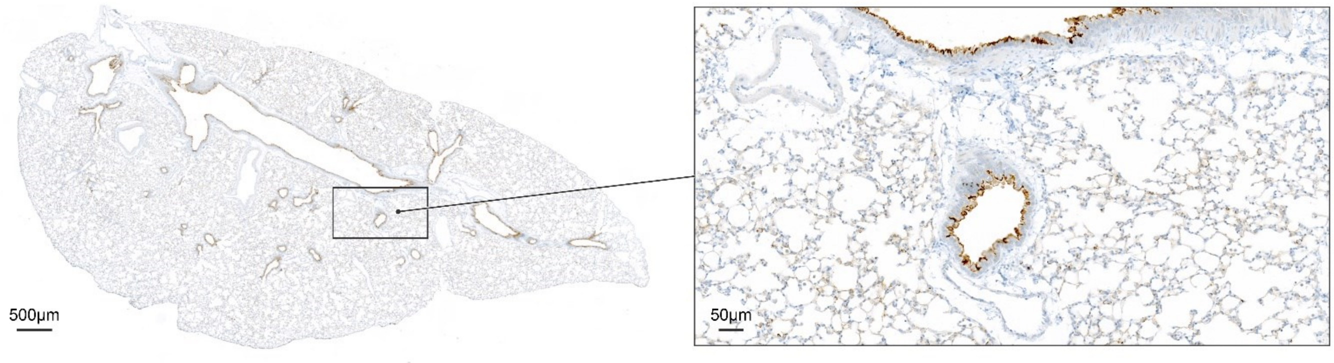

Supplementary Figure 4. Pulmonary viral burden. The viral load in the lungs of infected treated animals, represented as the total SARS-CoV-2 N protein copies, had a 1.46 log_{10} reduction compared to infected untreated controls (Mann-Whitney *U* test, two-tailed, *P*=0.1308). Data not normalized to *Pol2Ra*. Data from two independent experiments, infected untreated (n=11), infected treated (n=9).

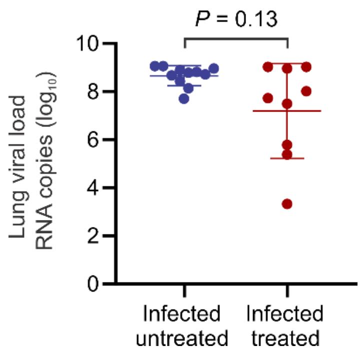

Supplementary Figure 5. Histology and SARS-CoV-2 spike protein immunostaining. Low magnification (scale bar, 1 mm) and the corresponding high magnification areas (scale bar, 50 μm) of Hematoxylin and eosin staining (top panels) and SARS-CoV-2 spike protein immunostaining (bottom panels) of the lungs of (**a**) uninfected, (**b**) infected SFN-treated, and (**c**) infected untreated animals.

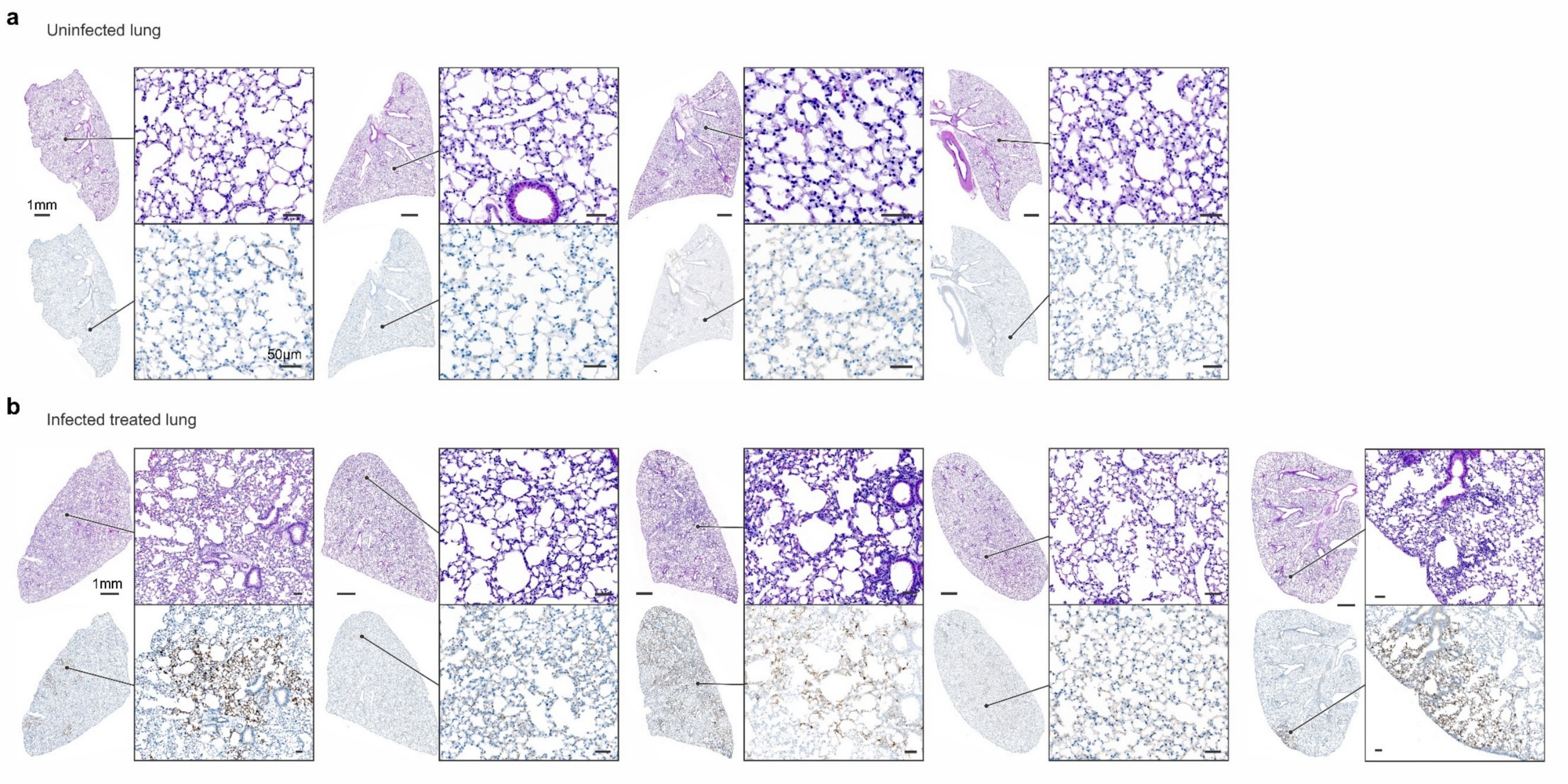

c

Infected untreated lung

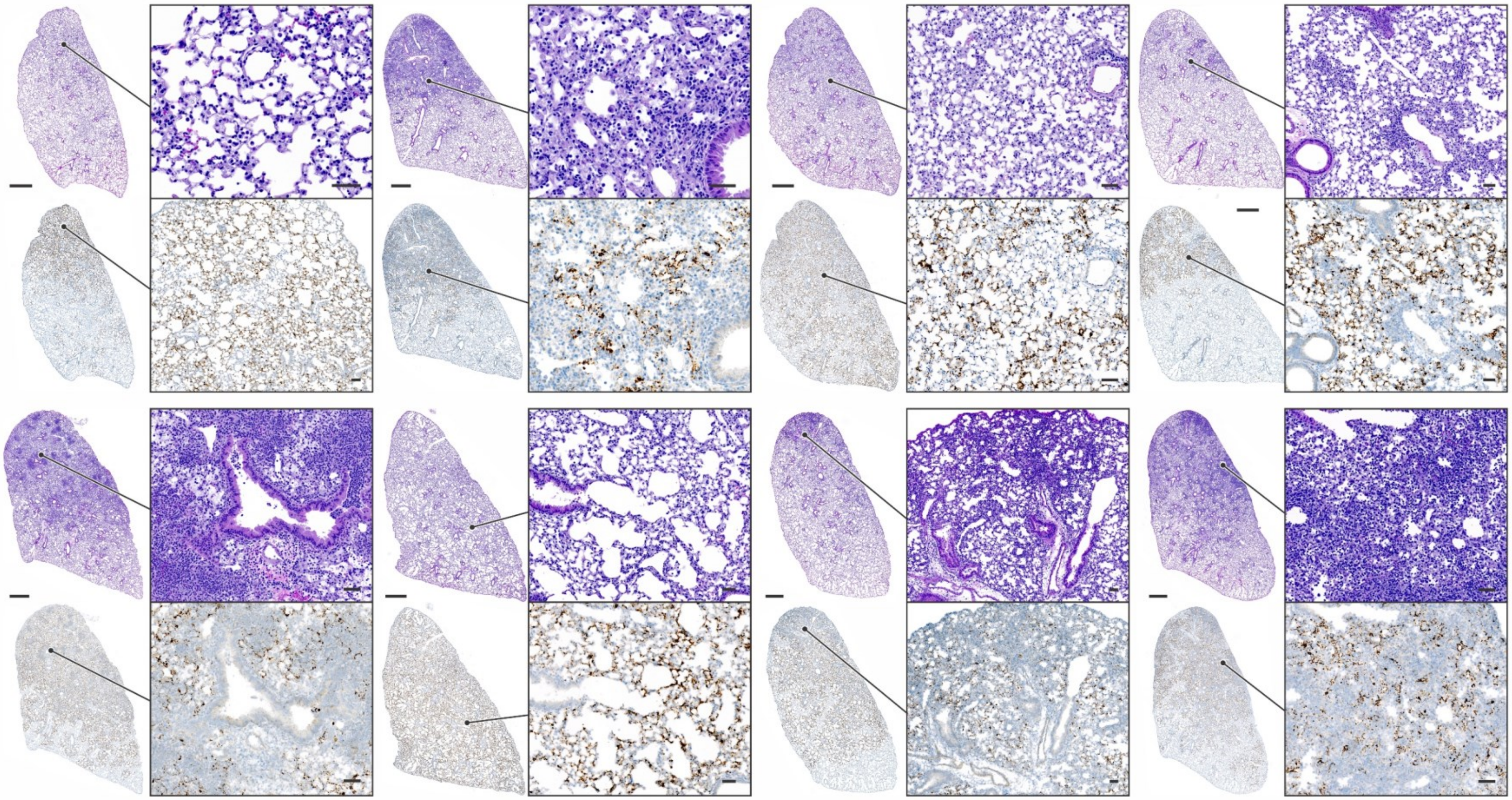

Supplementary Figure 6. Flow cytometric gating strategy. Flow cytometric gating strategy for macrophages (**a**), T cells (**b**), and T cell functional analysis (**c**), in lung, spleen, and bronchoalveolar lavage.

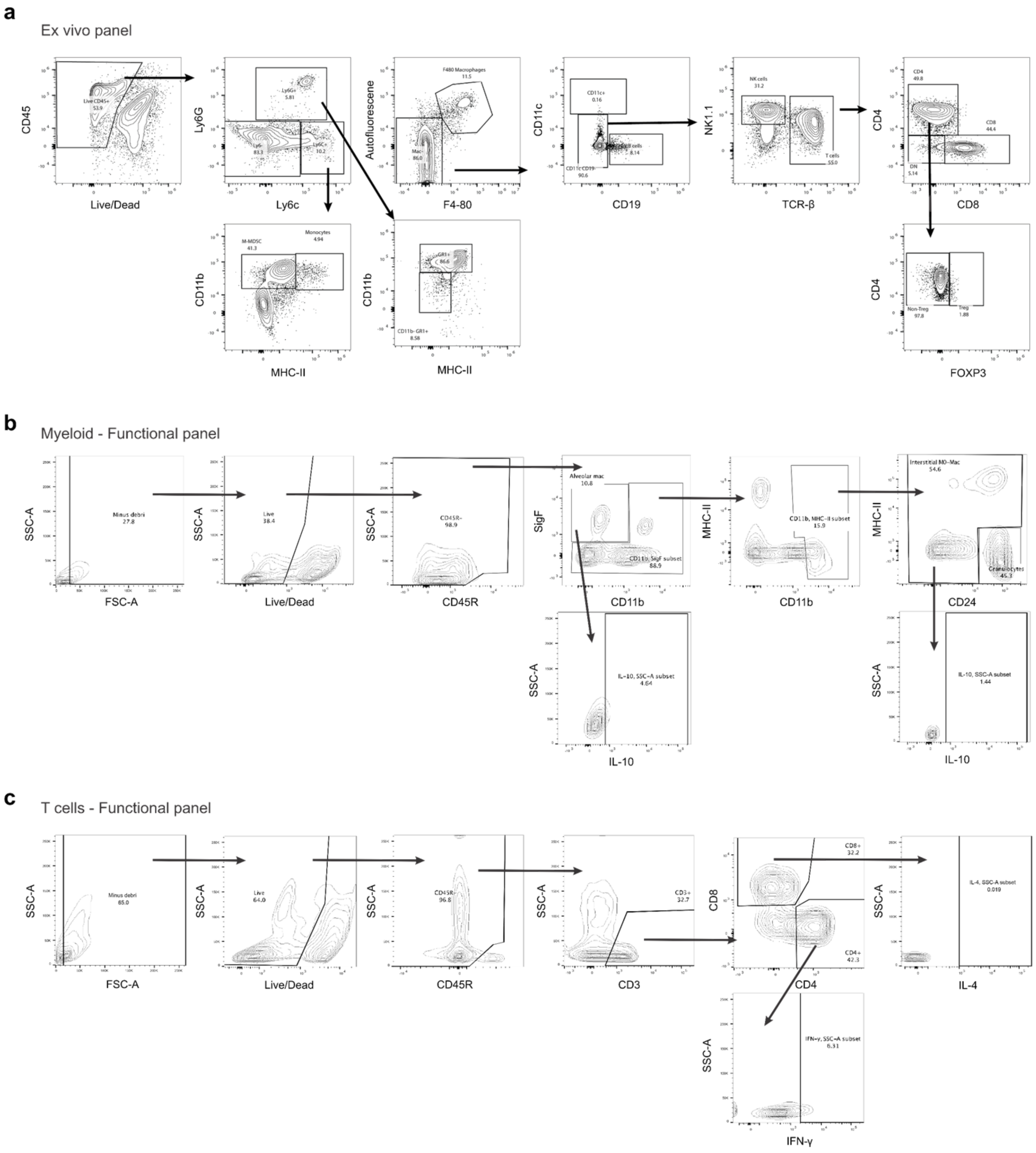

Supplementary Figure 7. Functional markers of immune response in the lung. Flow cytometric analysis of pulmonary alveolar macrophages (**a**) and interstitial macrophages (**b**). MFI, mean fluorescent intensity. Data represented as mean ± standard error of mean. n=4 uninfected, n=5 infected SFN-treated, and n=8 infected untreated animals. Statistical comparisons made with one-way ANOVA, *P<0.05, **P < 0.01, ***P < 0.001.

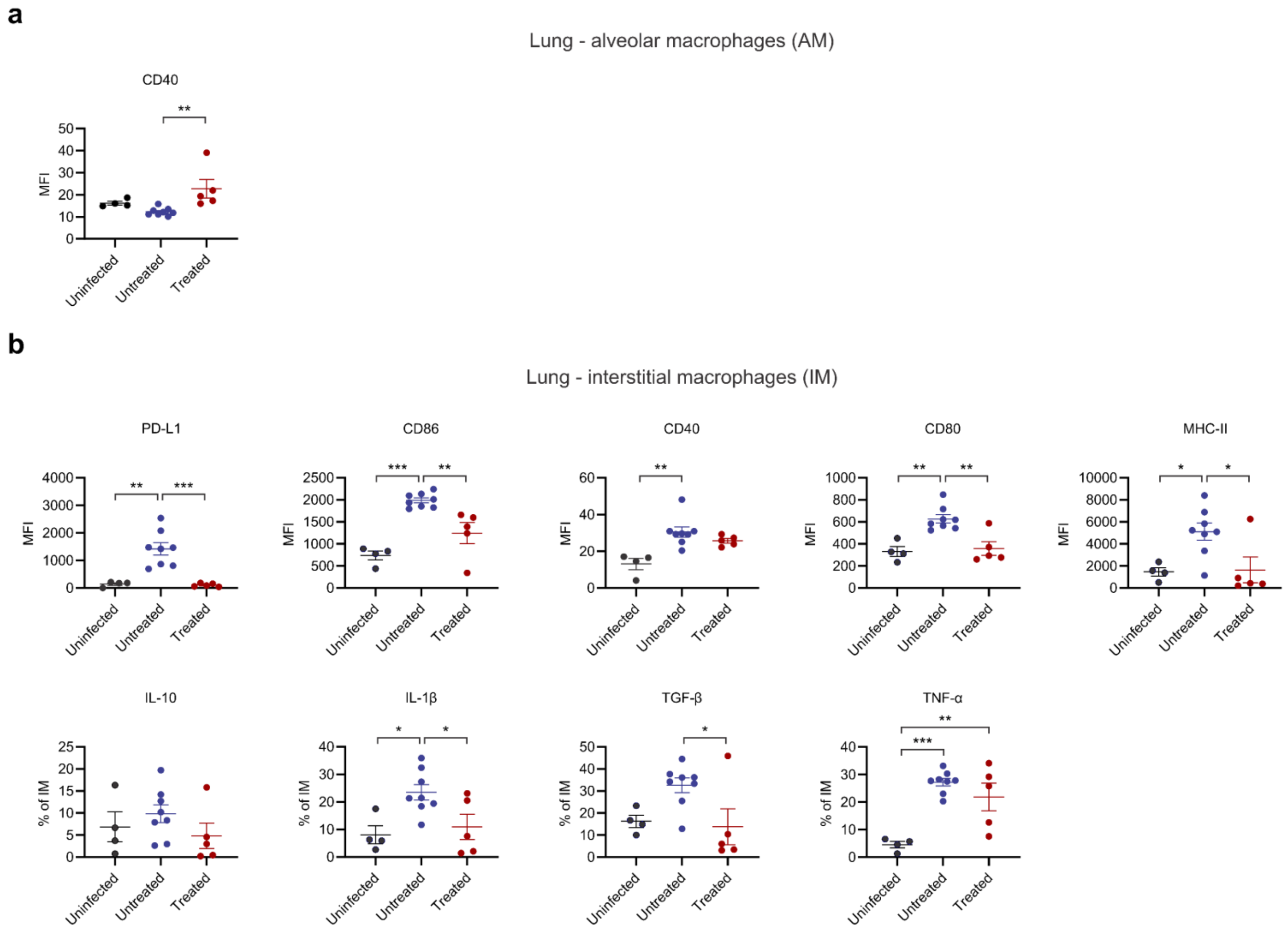

Supplementary Figure 8. Functional markers of macrophages in the bronchoalveolar lavage. Flow cytometric analysis of alveolar macrophages (**a**) and interstitial macrophages (**b**) of the bronchoalveolar lavage. MFI, mean fluorescent intensity. Data represented as mean ± standard error of mean. n=4 uninfected, n=5 infected SFN-treated, and n=8 infected untreated animals. Statistical comparisons made with one-way ANOVA, *P<0.05, **P < 0.01, ***P < 0.001.

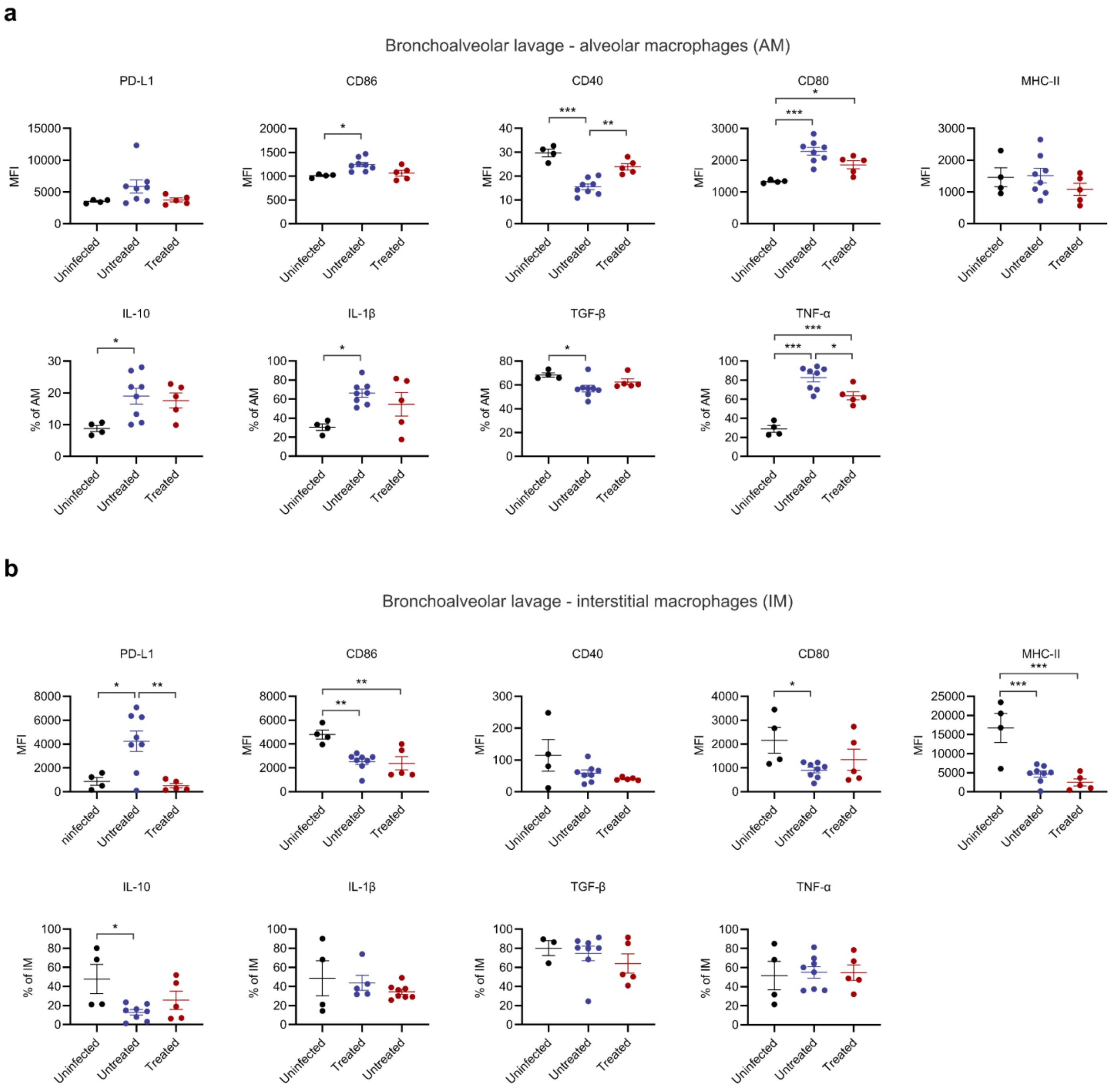

Supplementary Figure 9. Functional characterization of T cells. Flow cytometric analysis of T cells after stimulation with PMA/ionomycin in the spleen (**a**), lung (**b**), and bronchoalveolar lavage (**c**). MFI, mean fluorescent intensity. Data represented as mean ± standard error of mean. n=4 uninfected, n=5 infected SFN-treated, and n=8 infected untreated animals. Statistical comparisons made with one-way ANOVA, **P*<0.05, ***P* < 0.01, ****P* < 0.001, *****P* < 0.0001.

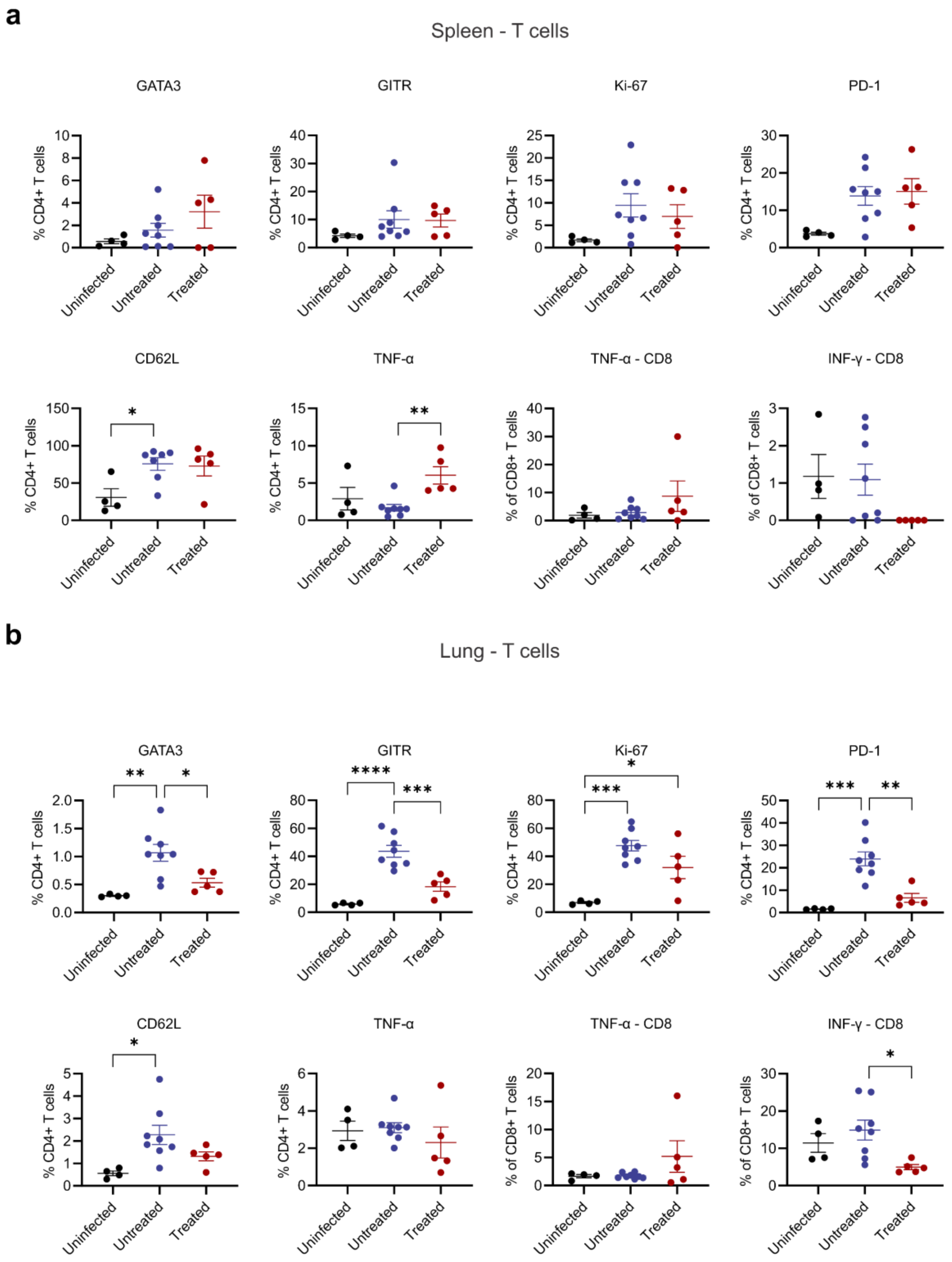

c

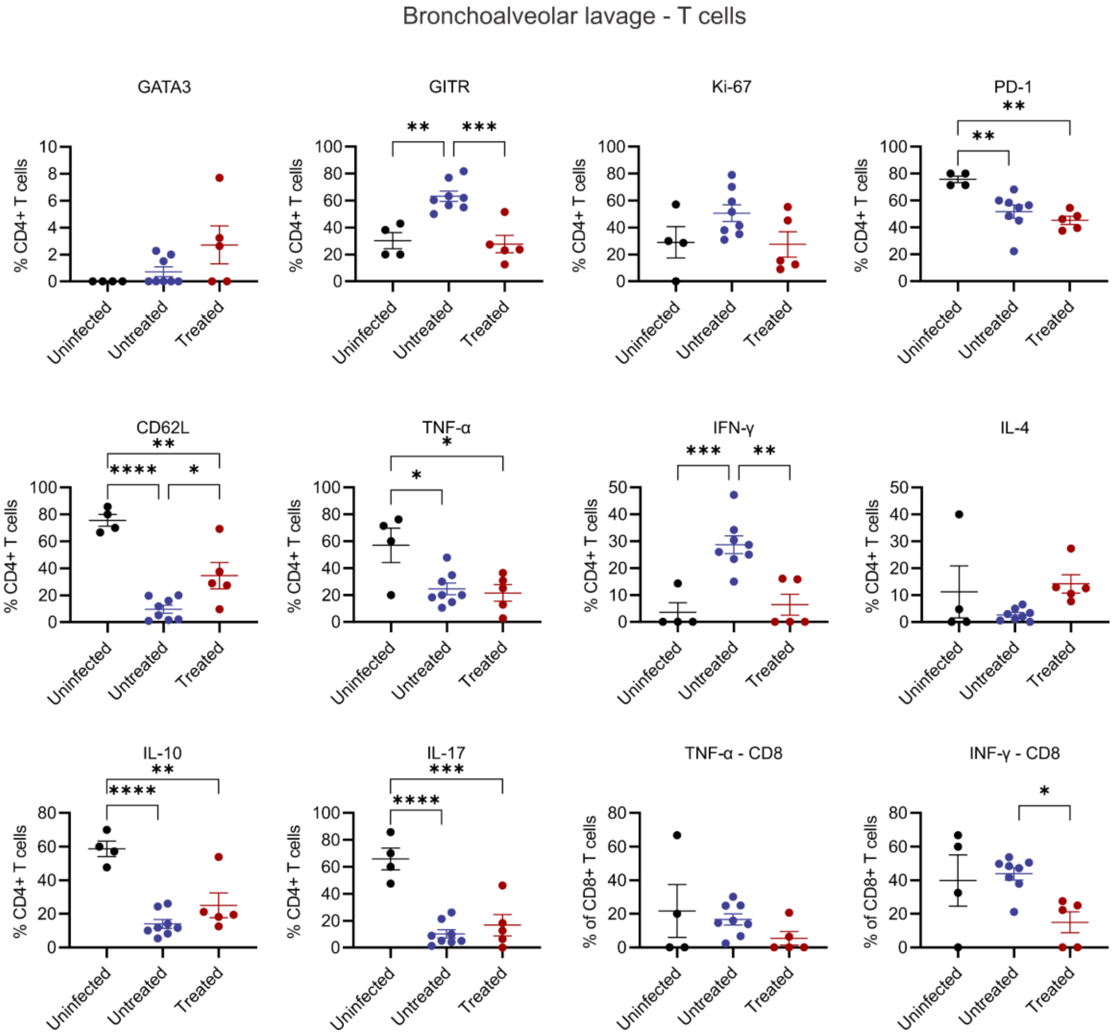
Bronchoalveolar lavage - T cells
GATA3
GITR
Ki-67
PD-1
CD62L
TNF-α
IFN-γ
IL-4
IL-10
IL-17
TNF-α - CD8
INF-γ - CD8
% CD4+ T cells
% of CD8+ T cells
Uninfected
Untreated
Treated

Supplementary Table 1. Antiviral effects of SFN against HCoV-OC43.

Panel	Cell line	Drug[a] (range tested, μM)	Time of drug addition	Time of virus inoculation	IC_{50} [b] (μM)	TD_{50} [b] (μM)	TI [b]
A	Vero C1008	SFN (320 – 0.032)	1 – 2 h before virus	1 - 2 h after drug	10	73	7
B	MRC-5	SFN (320 – 0.032)	1 – 2 h before virus	1 - 2 h after drug	18	83	5
C	Vero C1008	SFN (320 – 0.032)	24 h after virus	24 h before drug	18	88	5
D	Vero C1008	SFN (100 – 0.01)	24 h before virus	24 h after drug: drug washed out and then virus added	21	91	4
E	Vero C1008	RDV (100 – 0.01)	1 – 2 h before virus	1 - 2 h after drug	22	142	6
F	Vero C1008	SFN (100 – 0.01) L to R[c]<br>RDV (32 – 0.032) T to B	1 – 2 h before virus	1 - 2 h after drug	N/A[d]	N/A[d]	N/A[d]

[a] SFN, Sulforaphane; RDV, Remdesivir
[b] IC_{50}, Median inhibitory concentration; TD_{50}, Median cytotoxic dose; TI, Therapeutic index
[c] SFN diluted across plate, Left to Right (L to R); RDV diluted down plate, Top to Bottom (T to B)
[d] N/A, Not applicable. See Figure 1 and Results for Combination Index (CI) results

Supplementary Table 2. List of evaluated cell lines.

Cell line*	Source (Catalog number)	Organism	Tissue	Morphology	Disease
HCT-8 [HRT-18]	ATCC# (CCL-244)	Human	Colon	Epithelial	Adenocarcinoma
Caco-2	ATCC (HTB-37)	Human	Colon	Epithelial	Adenocarcinoma
MRC-5	ATCC (CCL-171)	Human	Lung	Fibroblast	Normal
Vero C1008 [Vero 76, clone E6, Vero E6]	ATCC (CRL-1586)	African green monkey	Kidney	Epithelial	Normal

*None of the cell lines listed are registered as a misidentified cell line according to the International Cell Line Authentication Committee (ICLAC) Register of Misidentified Cell Lines, version 11. https://iclac.org/databases/cross-contaminations/

#ATCC, American Type Culture Collection.

Supplementary Table 3. List of antibodies used for flow cytometry.

Marker	Fluorophore	Vendor	Catalog #	Clone
**Ex vivo panel**				
NK1.1	PE CF594	BD Biosciences	562864	PK136
CD19	PE Cy5	BioLegend	115510	6D5
CD62L	PECy7	BD Biosciences	560516	MEL-14
CD11b	AF700	BioLegend	101222	M1/70
CD4	APC Cy7	BD Biosciences	565650	RM4-5
Ly6G	efluor 450	ThermoFisher	48-5931-82	RB6-8C5
CD103	BV480	BD Biosciences	566201	M290
CD44	BV510	BioLegend	103044	IM7
Ly6c	BV570	BioLegend	128030	HK1.4
PD1	BV605	BioLegend	135220	29F.1A12
TCRb	BV650	BioLegend	109251	H57-597
MCHII	BV711	BioLegend	107643	M5/114.15.2
CD11c	BV750	BioLegend	117357	N418
F4/80	BV785	BioLegend	123141	BM8
CD69	BUV737	BD Biosciences	612793	H1.2F
CD8	BUV805	BD Biosciences	612898	53-6.7
CD45	Super Bright 436	Fischer Scientific	62045182	30-F11
CPT1a	AF488	Abcam	ab171449	8F6AE9
VDAC1	AF532	Abcam	ab14734	20B12AF2
H3K27Me3	PE	CST	40724	C36B11
FOXP3	PerCpCy5.5	ThermoFisher	45-5773-82	FJK-16x
Ki67	PerCp-eFluor710	ThermoFisher	46-5698-82	SolA15
GLUT1	AF647	Abcam	ab195020	EPR3915
Hexokinase II	Dylight680	Abcam	ab228819	EPR20839
Tomm20	AF405	Abcam	ab210047	EPR15581-54
**Macrophage panel**				
CD80	FITC	BioLegend	104716	16-10A1
CD11B	PE-CF594	ThermoFisher	RM2817	M1/70.15
CD64	PE-Cy7	BioLegend	139314	X54-5/7.1
CCR2	APC	BioLegend	150604	SA203G11
MHC-II	APC-Cy7	BioLegend	107628	M5/114.15.2
CD11C	APC-R700	BD Biosciences	565872	N418
LY6C	BV605	BioLegend	128036	HK1.4
CD86	BV650	BD Biosciences	564200	GL1
CD40	BV786	BD Biosciences	740891	3/23
CD45R-B	BV750	BioLegend	103261	RA3-6B2
B7-H1	BV711	BD Biosciences	563369	MIH5
CD24	BUV737	BD Biosciences	565308	M1/69
SigF	BUV395	BD Biosciences	740280	E50-2440
IL-10	PE	BioLegend	505008	JES5-16E3
IL-1B	PerCP	ThermoFisher	46-7114-82	NJTEN3
TGF-B	BV421	BD Biosciences	565638	TW7-16B4
TNF-a	BV510	BD Biosciences	563386	MP6-XT22
**T cell panel**				
CD62L	APC-R700	BD biosciences	565159	MEL-14
TCR	APC-750	BioLegend	109246	H57-597
PD-1	BV605	BioLegend	135220	29F.1A12
CD25	BV650	BD Biosciences	564021	PC61
CD357 (GITR)	BV711	BD Biosciences	563390	DTA-1
CD45R-B	BV750	BioLegend	103261	RA3-6B2
CD3	BV785	BioLegend	100355	145-2C11
CD4	BUV395	BD Biosciences	563790	GK1.5
CD8	BUV737	BD Biosciences	612759	53-6.7
IL-17A	AF 488	BioLegend	506910	TC11-18H10.1
IL-10	PE	BioLegend	505008	JES5-16E3
Ki-67	PerCPe710	ThermoFisher	46-5698-82	SolA15
IFN-y	PE-Cy7	BioLegend	505826	XMG1.2
GATA3	PE-CF594	BD Biosciences	563510	L50-823
FOXP3	APC	ThermoFisher	17-5773-82	FJK-16s
