# Supplementary material for: Sulforaphane exhibits antiviral activity against pandemic SARS-CoV-2 and seasonal HCoV-OC43 coronaviruses in vitro and in mice
Source: Commun Biol. 2022 Mar 18;5:242. doi: 10.1038/s42003-022-03189-z (PMC8933402; doi:10.1038/s42003-022-03189-z)
Supplement: Supplementary file 2 — Description of Additional Supplementary Files [file 42003_2022_3189_MOESM2_ESM.pdf]

# Description of Additional Supplementary Files

**File name:** Supplementary Data 1
**Description:** Source data underlying the graphs presented in the main and supplementary figures.
