# Supplementary material for: Sulforaphane exhibits antiviral activity against pandemic SARS-CoV-2 and seasonal HCoV-OC43 coronaviruses in vitro and in mice
Source: Commun Biol. 2022 Mar 18;5:242. doi: 10.1038/s42003-022-03189-z (PMC8933402; doi:10.1038/s42003-022-03189-z)
Supplement: Supplementary file 4 — Reporting Summary [file 42003_2022_3189_MOESM4_ESM.pdf]

Corresponding author(s): Lorraine Jones-Brando, Alvaro A. Ordonez

Last updated by author(s): Jan 14, 2022

Reporting Summary

Nature Research wishes to improve the reproducibility of the work that we publish. This form provides structure for consistency and transparency in reporting. For further information on Nature Research policies, see our Editorial Policies and the Editorial Policy Checklist.

Statistics

For all statistical analyses, confirm that the following items are present in the figure legend, table legend, main text, or Methods section.

n/a	Confirmed	
☐	☒	The exact sample size (*n*) for each experimental group/condition, given as a discrete number and unit of measurement
☐	☒	A statement on whether measurements were taken from distinct samples or whether the same sample was measured repeatedly
☐	☒	The statistical test(s) used AND whether they are one- or two-sided<br>*Only common tests should be described solely by name; describe more complex techniques in the Methods section.*
☒	☐	A description of all covariates tested
☐	☒	A description of any assumptions or corrections, such as tests of normality and adjustment for multiple comparisons
☐	☒	A full description of the statistical parameters including central tendency (e.g. means) or other basic estimates (e.g. regression coefficient) AND variation (e.g. standard deviation) or associated estimates of uncertainty (e.g. confidence intervals)
☐	☒	For null hypothesis testing, the test statistic (e.g. *F*, *t*, *r*) with confidence intervals, effect sizes, degrees of freedom and *P* value noted<br>*Give P values as exact values whenever suitable.*
☒	☐	For Bayesian analysis, information on the choice of priors and Markov chain Monte Carlo settings
☒	☐	For hierarchical and complex designs, identification of the appropriate level for tests and full reporting of outcomes
☒	☐	Estimates of effect sizes (e.g. Cohen's *d*, Pearson's *r*), indicating how they were calculated

Our web collection on statistics for biologists contains articles on many of the points above.

Software and code

Policy information about availability of computer code

Data collection	Excel 2019 (Microsoft); FACSAria II spectral flow cytometer (BD); Aurora spectral flow cytometer (Cytek); StepOne Plus Real Time PCR (Applied Biosystems); SoftMax Pro 6.5 (Molecular Devices); FLUOstar Omega plate reader (BMG Labtech).
Data analysis	Prism 9.0.1 (GraphPad); Halo v.3.2.1851.328 (Indica Labs), Flowjo v10.6.2 (BD); Calcusyn software (Biosoft).

For manuscripts utilizing custom algorithms or software that are central to the research but not yet described in published literature, software must be made available to editors and reviewers. We strongly encourage code deposition in a community repository (e.g. GitHub). See the Nature Research guidelines for submitting code & software for further information.

Data

Policy information about availability of data

All manuscripts must include a data availability statement. This statement should provide the following information, where applicable:

- Accession codes, unique identifiers, or web links for publicly available datasets
- A list of figures that have associated raw data
- A description of any restrictions on data availability

All the data supporting the findings of this study are found within the manuscript and its supplementary data, and are available from the corresponding author upon request.

Field-specific reporting

Please select the one below that is the best fit for your research. If you are not sure, read the appropriate sections before making your selection.

☒ Life sciences ☐ Behavioural & social sciences ☐ Ecological, evolutionary & environmental sciences

For a reference copy of the document with all sections, see nature.com/documents/nr-reporting-summary-flat.pdf

Life sciences study design

All studies must disclose on these points even when the disclosure is negative.

Sample size	No sample-size calculations were performed to power each study. Based on prior mouse studies with similar in vivo experiments, a sample size of 5-8 mice per group was determined as minimally sufficient to produce a study power of >80% (PMID: 32553273, 32839612).
Data exclusions	One animal from the uninfected group was excluded from all analyses due to skin injuries caused by aggression from other mice in the same cage. No other animals were excluded.
Replication	All the experiments were subjected to at least three biological replicates unless otherwise specified.
Randomization	Mice were randomly assigned to each group (uninfected, infected untreated, infected SFN-treated).
Blinding	The investigators were not blinded to allocation during experiments and outcome assessment. For scoring of histopathology, the histological slides were blinded prior to scoring.

Reporting for specific materials, systems and methods

We require information from authors about some types of materials, experimental systems and methods used in many studies. Here, indicate whether each material, system or method listed is relevant to your study. If you are not sure if a list item applies to your research, read the appropriate section before selecting a response.

Materials & experimental systems

n/a	Involved in the study	
☐	☒	Antibodies
☐	☒	Eukaryotic cell lines
☒	☐	Palaeontology and archaeology
☐	☒	Animals and other organisms
☒	☐	Human research participants
☒	☐	Clinical data
☒	☐	Dual use research of concern

Methods

n/a	Involved in the study	
☒	☐	ChIP-seq
☐	☒	Flow cytometry
☒	☐	MRI-based neuroimaging

Antibodies

Antibodies used	NK1.1 (PE CF594) - BD Biosciences catalog # 562864, clone PK136. CD19 (PE Cy5) - BioLegend catalog # 115510, clone 6D5. CD62L (PECy7) - BD Biosciences catalog # 560516, clone MEL-14. CD11b (AF700) - BioLegend catalog # 101222, close M1/70. CD4 (APC Cy7) - BD Biosciences catalog # 565650, clone RM4-5. Ly6G (efluor 450) – ThermoFisher catalog # 48-5931-82, clone RB6-8C5. CD103 (BV480) - BD Biosciences catalog # 566201, clone M290. CD44 (BV510) – BioLegend catalog # 103044, clone IM7. Ly6c (BV570) – BioLegend catalog # 128030, clone HK1.4. PD1 (BV605) – BioLegend catalog # 135220, clone 29F.1A12. TCRb (BV650) – BioLegend catalog # 109251, clone H57-597. MCHII (BV711) – BioLegend catalog # 107643, clone M5/114.15.2. CD11c (BV750) – BioLegend catalog # 117357, clone N418. F4/80 (BV785) - BioLegend catalog # 123141, clone BM8. CD69 (BUV737) - BD Biosciences catalog # 612793, clone H1.2F. CD8 (BUV805) - BD Biosciences catalog # 612898, clone 53-6.7. CD45 (Super Bright 436) - Fischer Scientific catalog # 62045182, clone 30-F11. CPT1a (AF488) – Abcam catalog # ab171449, clone 8F6AE9. VDAC1 (AF532) – Abcam catalog # ab14734, clone 20B12AF2. H3K27Me3 (PE) – CST catalog # 40724, clone C36B11. FOXP3 (PerCpCy5.5) – ThermoFisher catalog # 45-5773-82, clone FJK-16x. Ki67 (PerCp-eFluor710) – ThermoFisher catalog # 46-5698-82, clone SolA15. GLUT1 (AF647) - Abcam catalog # ab195020, clone EPR3915. Hexokinase II (Dylight680) – Abcam catalog # ab228819, clone EPR20839. Tomm20 (AF405) – Abcam catalog # ab210047, clone EPR15581-54. CD80 (FITC) – BioLegend catalog # 104716, clone 16-10A1. CD11B (PE-CF594) – ThermoFisher catalog # RM2817, clone M1/70.15. CD64 (PE-Cy7) – BioLegend catalog # 139314, clone X54-5/7.1. CCR2 (APC) – BioLegend catalog # 150604, clone SA203G11. MHC-II (APC-Cy7) – BioLegend catalog # 107628, clone M5/114.15.2. CD11C (APC-R700) - BD Biosciences catalog # 565872, clone N418. LY6C (BV605) – BioLegend catalog # 128036, clone HK1.4. CD86 (BV650) - BD Biosciences catalog # 564200G, clone GL1. CD40 (BV786) -BD Biosciences catalog # 740891, clone 3/23. CD45R-B (BV750) – BioLegend catalog # 103261, clone RA3-6B2. B7-H1 (BV711) - BD Biosciences catalog # 563369, clone MIH5. CD24 (BUV737) - BD Biosciences catalog # 565308, clone M1/69. SigF (BUV395) - BD Biosciences catalog # 740280, clone E50-2440. IL-10 (PE) – BioLegend catalog # 505008, clone JES5-16E3. IL-1B (PerCP) – ThermoFisher catalog # 46-7114-82, clone NJTEN3. TGF-B (BV421) - BD Biosciences catalog # 565638, clone TW7-16B4. TNF-a (BV510) - BD Biosciences catalog # 563386, clone MP6-XT22. CD62L (APC-R700) - BD biosciences catalog # 565159, clone MEL-14. TCR (APC-750) – BioLegend catalog # 109246, clone H57-597. PD-1 (BV605)

– BioLegend catalog # 135220, clone 29F.1A12. CD25 (BV650) - BD Biosciences catalog # 564021, clone PC61. CD357 - GITR (BV711) - BD Biosciences catalog # 563390, clone DTA-1. CD45R-B (BV750) – BioLegend catalog # 103261, clone RA3-6B2. CD3 (BV785) – BioLegend catalog # 100355, clone 145-2C11. CD4 (BUV395) - BD Biosciences catalog # 563790, clone GK1.5. CD8 (BUV737) - BD Biosciences catalog # 612759, clone 53-6.7. IL-17A (AF 488) – BioLegend catalog # 506910, clone TC11-18H10.1. IL-10 (PE) – BioLegend catalog # 505008, clone JES5-16E3. Ki-67 (PerCPe710) – ThermoFisher catalog # 46-5698-82, clone SolA15. IFN-y (PE-Cy7) – BioLegend catalog # 505826, clone XMG1.2. GATA3 (PE-CF594) - BD Biosciences catalog # 563510, clone L50-823. FOXP3 (APC) – ThermoFisher catalog # 17-5773-82, clone FJK-16s. Anti-SARS-CoV-2 spike protein – Genetex catalog # GTX135356, lot # 43957.Recombinant Anti-Nrf2 antibody [EP1808Y] - Abcam catalog #ab62352.

Validation

For primary antibody validation, product citations on the manufacturer's website are listed below:

Ex vivo panel
1. NK1.1 (PE CF594) - BD Biosciences catalog # 562864, clone PK136. Reference: PMID 9396764
2. CD19 (PE Cy5) - BioLegend catalog # 115510, clone 6D5. Reference: PMID 29426701
3. CD62L (PE Cy7) - BD Biosciences catalog # 560516, clone MEL-14. Reference: PMID 6866086
4. CD11b (AF700) - BioLegend catalog # 101222, clone M1/70. Reference: PMID 30012638
5. CD4 (APC Cy7) - BD Biosciences catalog # 565650, clone RM4-5. Reference: PMID 1836198
6. Ly6G (efluor 450) – ThermoFisher catalog # 48-5931-82, clone RB6-8C5. Reference: PMID 31285854
7. CD103 (BV480) - BD Biosciences catalog # 566201, clone M290. Reference: PMID 7705417
8. CD44 (BV510) – BioLegend catalog # 103044, clone IM7. Reference: PMID 29606612
9. Ly6c (BV570) – BioLegend catalog # 128030, clone HK1.4. Reference: PMID 29958801
10. PD1 (BV605) – BioLegend catalog # 135220, clone 29F.1A12. Reference: PMID 29963060
11. TCRb (BV650) – BioLegend catalog # 109251, clone H57-597. Reference: PMID 17702899
12. MCHII (BV711) – BioLegend catalog # 107643, clone M5/114.15.2. Reference: PMID 30553275
13. CD11c (BV750) – BioLegend catalog # 117357, clone N418. Reference: PMID 19786541
14. F4/80 (BV785) - BioLegend catalog # 123141, clone BM8. Reference: PMID 29858009
15. CD69 (BUV737) - BD Biosciences catalog # 612793, clone H1.2F. Reference: PMID 10550051
16. CD8 (BUV805) - BD Biosciences catalog # 612898, clone 53-6.7. Reference: PMID 8609387
17. CD45 (Super Bright 436) - Fisher Scientific catalog # 62045182, clone 30-F11. Reference: PMID 30970240
18. CPT1a (AF488) - Abcam catalog # ab171449, clone 8F6AE9. Reference: PMID 29415897
19. VDAC1 (AF532) – Abcam catalog # ab14734, clone 20B12AF2. Reference: PMID AF488
20. H3K27Me3 (PE) – CST catalog # 40724, clone C36B11. Reference: PMID 33691089
21. FOXP3 (PerCpCy5.5) – ThermoFisher catalog # 45-5773-82, clone FJK-16s. Reference: PMID 31940493
22. Ki67 (PerCp-eFluor710) – ThermoFisher catalog # 46-5698-82, clone SolA15. Reference: PMID 27999749
23. GLUT1 (AF647) - Abcam catalog # ab195020, clone EPR3915. Reference: PMID 29884706
24. Hexokinase II (Dylight680) – Abcam catalog # ab228819, clone EPR20839. Reference: PMID 32537015
25. Tomm20 (AF405) – Abcam catalog # ab210047, clone EPR15581-54. Reference: PMID 30847435

Macrophage panel
1. CD80 (FITC) - BioLegend catalog # 104716, clone 16-10A1. Reference: PMID 31216480
2. CD11B (PE-CF594) – ThermoFisher catalog # RM2817, clone M1/70.15. Reference: PMID 28683285
3. CD64 (PE-Cy7) – BioLegend catalog # 139314, clone X54-5/7.1. Reference: PMID 29958798
4. CCR2 (APC) – BioLegend catalog # 150604, clone SA203G11. Reference: PMID 31581150
5. MHC-II (APC-Cy7) – BioLegend catalog # 107628, clone M5/114.15.2. Reference: PMID 31101805
6. CD11C (APC-R700) - BD Biosciences catalog # 565872, clone N418. Reference: PMID 2145370
7. LY6C (BV605) – BioLegend catalog # 128036, clone HK1.4. Reference: PMID 30266340
8. CD86 (BV650) - BD Biosciences catalog # 564200 , clone GL1. Reference: PMID 9075931
9. CD40 (BV786) - BD Biosciences catalog # 740891, clone 3/23. Reference: PMID 7519998
10. CD45R-B (BV750) – BioLegend catalog # 103261, clone RA3-6B2. Reference: PMID 18077787
11. B7-H1 (BV711) - BD Biosciences catalog # 563369, clone MIH5. Reference: PMID 16314434
12. CD24 (BUV737) - BD Biosciences catalog # 565308, clone M1/69. Reference: PMID 81133
13. SigF (BUV395) - BD Biosciences catalog # 740280, clone E50-2440. Reference: PMID 11579105
14. IL-10 (PE) – BioLegend catalog # 505008, clone JES5-16E3. Reference: PMID 30611611
15. IL-1B (PerCP) – ThermoFisher catalog # 46-7114-82, clone NJTEN3. Reference: PMID 24336249
16. TGF-B (BV421) - BD Biosciences catalog # 565638, clone TW7-16B4. Reference: PMID 20720212
17. TNF-a (BV510) - BD Biosciences catalog # 563386, clone MP6-XT22. Reference: PMID 18432811

T cell panel
1. CD62L (APC-R700) - BD biosciences catalog # 565159, clone MEL-14. Reference: PMID 7687616
2. TCR (APC-750) – BioLegend catalog # 109246, clone H57-597. Reference: PMID 17702899
3. PD-1 (BV605) – BioLegend catalog # 135220, clone 29F.1A12. Reference: PMID 29963060
4. CD25 (BV650) - BD Biosciences catalog # 564021, clone PC61. Reference: PMID 2136898
5. CD357 - GITR (BV711) - BD Biosciences catalog # 563390, clone DTA-1. Reference: PMID 12847237
6. CD45R-B (BV750) – BioLegend catalog # 103261, clone RA3-6B2. Reference: PMID 18077787
7. CD3 (BV785) – BioLegend catalog # 100355, clone 145-2C11. Reference: PMID 30659197
8. CD4 (BUV395) - BD Biosciences catalog # 563790, clone GK1.5. Reference: PMID 6415170
9. CD8 (BUV737) - BD Biosciences catalog # 612759, clone 53-6.7. Reference: PMID 8609387
10. IL-17A (AF 488) – BioLegend catalog # 506910, clone TC11-18H10.1. Reference: PMID 30282028
11. IL-10 (PE) – BioLegend catalog # 505008, clone JES5-16E3. Reference: PMID 30611611
12. Ki-67 (PerCPe710) – ThermoFisher catalog # 46-5698-82, clone SolA15. Reference: PMID 27999749
13. IFN-y (PE-Cy7) – BioLegend catalog # 505826, clone XMG1.2. Reference: PMID 29127360
14. GATA3 (PE-CF594) - BD Biosciences catalog # 563510, clone L50-823. Reference: PMID 21358638
15. FOXP3 (APC) – ThermoFisher catalog # 17-5773-82, clone FJK-16s. Reference: PMID 29441059

Immunohistochemistry
1. Anti-SARS-CoV-2 spike protein – Genetex catalog # GTX135356, lot # 43957. Reference: PMID 33631118

Western blot
1. Recombinant Anti-Nrf2 antibody [EP1808Y] - Abcam catalog #ab62352. Reference: PMID 33441941

Eukaryotic cell lines

Policy information about cell lines

Cell line source(s)	All cells used in this study were obtained from the American Type Culture Collection (ATCC). HCT-8 [HRT-18] (ATCC Catalog # CCL-244); Vero C1008 [Vero 76, clone E6, Vero E6] (ATCC Catalog # CRL-1586); MRC-5 (ATCC Catalog # CCL-171); Caco-2 (ATCC Catalog # HTB-37). Additional details are provided in Supplementary Table 2.
Authentication	The cell lines used in this study were not authenticated since they were purchased from commercial source and were extensively validated before shipping. Morphology for each cell line was assessed by microscope. Cell lines were always handled separately in laminar flow biosafety cabinets with at least 15 minutes of clearing between different lines. All media for each cell line is designated for that specific cell line and never used for other cell lines.
Mycoplasma contamination	The cell lines used in this study were not tested for mycoplasma contamination during the course of the study since they were 1) purchased from the ATCC and certified mycoplasma-negative and, 2) all media components were also purchased from commercial sources and certified mycoplasma-negative.
Commonly misidentified lines (See ICLAC register)	No cell lines used in this study are listed in ICLAC v10.

Animals and other organisms

Policy information about studies involving animals; ARRIVE guidelines recommended for reporting animal research

Laboratory animals	Heterozygous K18-hACE2 C57BL/6J mice (strain: 2B6.Cg-Tg(K18-ACE2)2Prlmn/J) were obtained from The Jackson Laboratory and propagated at Johns Hopkins University School of Medicine. Male mice, 6-8 weeks old were used for this study.
Wild animals	No wild animals were used in this study.
Field-collected samples	No field-collected samples were used in this study.
Ethics oversight	Animal studies were carried out based on the recommendations in the Guide for the Care and Use of Laboratory Animals of the National Institutes of Health. The protocols were approved by the Johns Hopkins University Institutional Animal Care and Use Committee.

Note that full information on the approval of the study protocol must also be provided in the manuscript.

Flow Cytometry

Plots

Confirm that:

☒ The axis labels state the marker and fluorochrome used (e.g. CD4-FITC).

☒ The axis scales are clearly visible. Include numbers along axes only for bottom left plot of group (a 'group' is an analysis of identical markers).

☒ All plots are contour plots with outliers or pseudocolor plots.

☒ A numerical value for number of cells or percentage (with statistics) is provided.

Methodology

Sample preparation	Lungs were minced and incubated at 37°C in an enzyme cocktail of RPMI containing 2.4 mg/ml collagenase I and 20 µg/ml DNase (Invitrogen), then mashed through a 70-µm nylon cell strainer (BD Falcon). All flow cytometry antibodies used for phenotypic and metabolic analysis can be found in Table S1. For analysis immediately ex vivo, cells were washed once in PBS and immediately stained for viability with Biolegend Live/Dead Zombie NIR Fixable Viability Dye and Fc Block for 10 min at room temperature. Cell surface staining was performed in 100µL of 20% BD HorizonTM Brilliant Stain Buffer + PBS with surface stain antibody cocktail for 20 min at room temperature. Cells were fixed and permeabilized with eBioscienceTM FoxP3/Transcription Factor Staining kit 1x Fixation/Permeabilization reagent overnight at 4°C. Cells were washed with 1x Permeabilization/Wash buffer. Intracellular staining (ICS) was performed in 100µL 1x Permeabilization/Wash buffer with ICS antibody cocktail for 45 min at room temperature. Cells were washed once with Permeabilization/Wash buffer then resuspended in Permeabilization/Wash buffer for acquisition by flow. To improve the quality of the T-cell flow cytometry functional staining, the cells were stimulated with phorbol 12-myristate 13-acetate (PMA, 50ng/mL) and inomycin (1µg/mL) for 1h, following for a 3h incubation with protein transport inhibitors (GolgiPlug and GolgiStop, BD). For the myeloid flow cytometry functional staining, the cells were incubated only with protein transport inhibitors for 4h.
Instrument	Aurora spectral flow cytometer (Cytek) and FACSAria II spectral flow cytometer (BD).

Software	Flowjo v10.6.2 (BD). Flowjo plugins DownSample v3 and UMAP were used for Uniform Manifold Approximation and Projection (UMAP) analyses.
Cell population abundance	No cell sorting was performed in this study.
Gating strategy	All gating strategies are shown in extended data figures.

☒ Tick this box to confirm that a figure exemplifying the gating strategy is provided in the Supplementary Information.
